# Regional differences in the impact of the COVID-19 pandemic on food sufficiency in California, April–July 2020: implications for food programmes and policies

**DOI:** 10.1017/S1368980021001889

**Published:** 2021-04-30

**Authors:** Evelyn Blumenberg, Miriam Pinski, Lilly A Nhan, May C Wang

**Affiliations:** 1 Department of Urban Planning, Luskin School of Public Affairs, University of California, Los Angeles, 3250 Public Affairs Building, Los Angeles, CA 90095, USA; 2 Department of Community Health Sciences, Fielding School of Public Health, University of California, Los Angeles, 650 Charles E Young Dr S, Los Angeles, CA 90095, USA

**Keywords:** Food insufficiency, COVID-19, Food policy, Health disparities

## Abstract

**Objective:**

To evaluate regional differences in factors associated with food insufficiency during the initial months of the COVID-19 pandemic among three major metropolitan regions in California, a state with historically low participation rates in the Supplementation Nutrition Assistance Program, the nation’s largest food assistance programme.

**Design:**

Analysis of cross-sectional data from phase 1 (23 April–21 July 2020) of the US Census Household Pulse Survey, a weekly national online survey.

**Setting:**

California, and three Californian metropolitan statistical areas (MSA), including San Francisco–Oakland–Berkeley, Los Angeles–Long Beach–Anaheim and Riverside–San Bernardino–Ontario MSA.

**Participants:**

Adults aged 18 years and older living in households.

**Results:**

Among the three metropolitan areas, food insufficiency rates were lowest in the San Francisco–Oakland–Berkeley MSA. Measures of disadvantage (e.g., having low-income, being unemployed, recent loss of employment income and pre-pandemic food insufficiency) were widely associated with household food insufficiency. However, disadvantaged households in the San Francisco Bay Area, the area with the lowest poverty and unemployment rates, were more likely to be food insufficient compared with those in the Los Angeles–Long Beach–Anaheim and Riverside–San Bernardino–Ontario MSA.

**Conclusions:**

Food insufficiency risk among disadvantaged households differed by region. To be effective, governmental response to food insufficiency must address the varied local circumstances that contribute to these disparities.

The COVID-19 pandemic has caused unprecedented disruptions to the social and economic livelihoods of Americans. While ‘safer-at-home’ (also known as ‘shelter-in-place’) and social distancing orders across the USA were important for mitigating the spread of COVID-19, these orders affected people’s ability to work and, consequently their financial resources for purchasing food. With the closure of non-essential businesses, millions of Americans experienced job and income losses during the pandemic. For families with children, the physical closure of schools and child care facilities limited their ability to receive food for their children through critical federal food assistance programmes such as school meals^(1)^. At the same time, the economic disruptions from COVID-19 coupled with shifts away from institutional and restaurant food consumption and towards consuming food at home caused shocks to the food system and resulted in increased food prices early during the pandemic^(2)^.

These rapid changes in employment and food access led to a rise in food insecurity, defined as ‘the limited or uncertain availability of nutritionally adequate and safe foods or limited or uncertain ability to acquire acceptable foods in socially acceptable ways’^(3)^. In high-income countries, such as the USA, food insecurity is associated with increased risk of diet-related chronic health conditions, such as obesity, diabetes and hypertension, and also with poor sleep and depression^(4)^. Hence, food insecurity, if not addressed promptly, can be expected to widen existing health disparities during the pandemic.

Early estimates suggest that nearly 40 % of US adults experienced food insecurity in the last week of March 2020^(5)^; another report estimated that 25 % of US adults experienced food insecurity from April to June 2020^(6)^. In comparison, the food insecurity rate for the nation was 10 % in 2019^(7)^. Patterns of food insecurity vary across population groups. Prior to the COVID-19 pandemic, food insecurity rates were highest among low-income, Black and Latinx households, and adults with children^(7)^, and these disparities have continued during the pandemic^(8)^.

In March 2020, Congress passed the Coronavirus Aid, Relief, and Economic Security (CARES) Act, which provided direct economic assistance to Americans, including stimulus payments of up to $1200 for eligible adults and $500 per qualifying child under age 17 years and expansion of unemployment benefits. Distribution of the first round of stimulus payments from the CARES Act started in April 2020. Early evidence suggests that stimulus payments and expansion of unemployment benefits through the CARES Act may have mitigated – but did not put an end to – food insecurity. For example, the Understanding the Coronavirus in America tracking survey reported that food insecurity in Los Angeles county, the most populous county in the USA, decreased from 26 % in April–May 2020 to 10 % in June–July 2020^(9)^. Among those who were food insecure in April–May 2020 but food secure in June–July 2020, 24·6 % received unemployment insurance and 17·2 % received economic stimulus funds. In comparison, among those who remained food insecure throughout April–July 2020, only 11·1 % received unemployment insurance and 11·6 % received economic stimulus funds. These findings suggest that financial assistance programmes may have assisted some families, but did not reach all families and did not provide sufficient relief.

Through the Census Household Pulse Survey (CHHPS), the Census Bureau has been measuring changes in food insufficiency during this pandemic, beginning data collection via the internet on 23 April 2020^(10)^. Food insufficiency – defined simply as having not enough food to eat – is considered a component of the broader concept of food insecurity^(3)^. Monitoring food insufficiency is important as it informs the implementation of programmes and policies designed to ensure that every household has access to enough food.

We used data from the CHHPS to examine regional differences in the determinants of food insufficiency in three diverse California metropolitan statistical areas (MSA): San Francisco–Oakland–Berkeley (SF-Bay Area), Los Angeles–Long Beach–Anaheim (LA-Anaheim) and Riverside–San Bernardino–Ontario (Riverside-San Bernardino). California was the first state to implement a statewide stay-at-home order (on 20 March 2020) and in the week of 12 September 2020, over 2·8 million people had claimed unemployment insurance in California, more than any other state^(11)^.

The effects of the COVID-19 pandemic on food insufficiency can be expected to vary across regions of the state, as the regions differ across many dimensions. For example, among the three MSA, LA-Anaheim and Riverside-San Bernardino had the highest unemployment rates (5·7 and 5·1 %, respectively), and the SF-Bay Area the lowest (3·5 %) in March 2020^(12)^. From March to May, unemployment rates increased substantially across the state. However, while May unemployment rates continued to remained higher in LA-Anaheim and Riverside-San Bernardino (19·1 and 14·9 %, respectively) than the SF-Bay Area (12·7 %), the percentage increase was largest in the SF-Bay Area^(13)^. There also are differences in inequality and the cost of living across the three MSA. Income inequality – measured as the ratio between the household income of those in the 95th percentile relative to those in the 20th percentile – is substantially higher in the SF-Bay Area than the other two metropolitan areas^(14)^. The cost of living in the SF-Bay Area is also the most expensive of the three regions^(15)^. Designed to understand regional differences in the determinants of food insufficiency, the current study will help inform the development of economic relief programmes during a crisis and guide the local delivery of services by federal food assistance programmes such as the Supplemental Nutrition Assistance Program (SNAP).

## Methods

We used microdata from the CHHPS, a weekly national survey designed to track the effects of the coronavirus pandemic on people’s lives and analysed data for California from phase 1 of data collection (12 weeks from 23 April 2020 to 21 July 2020)^(10)^. Respondents across all geographic areas in our analysis were surveyed in the same time periods for each week of data collection. The survey used the Census Bureau’s Master Address File to select a very large sample of housing units to allow for anticipated low response rates that could still yield estimates at the state and MSA levels. Sample sizes were determined to allow for a two-percentage point weekly change in key estimates to be detected at the national, state or MSA level with a 90 % CI. Sampled households were contacted to participate in the CHHPS via email or text if an email address was not available. During phase 1 of CHHPS, survey participants could complete the survey for up to three consecutive weeks of data collection^(16)^. The survey was administered online and gathered demographic, social, economic and health information, including assessment of food insufficiency prior to initial efforts to lock down the economy (referred to as ‘pre-pandemic’ for ease of reading) and at the time of the survey. The following question was used to assess household food insufficiency:

*Getting enough food can also be a problem for some people. Which of these statements best describes the food eaten in your household before March 13, 2020 [repeated for the ‘last 7 days’]? Select only one answer.*

- *Enough of the kinds of food (I/we) wanted to eat*
- *Enough, but not always the kinds of food (I/we) wanted to eat*
- *Sometimes not enough to eat*
- *Often not enough to eat*

Food insufficiency was operationally defined to include respondents who ‘sometimes’ or ‘often did not have enough to eat’.

We used logistic regression to evaluate the likelihood that a respondent was food insufficient. The models include socio-economic and demographic factors associated with food insufficiency, specifically, respondent age, sex, race/ethnicity, education and employment status; household income, household size, number of children and recent loss of income; pre-pandemic food insufficiency (not enough to eat prior to 13 March 2020); and survey week (week 1 to week 12, treated as an indicator variable). We built several models with the first for all California respondents testing MSA fixed effects to assess whether MSA differences remained after simultaneously controlling for confounding determinants of food insufficiency. We then stratified the data by MSA to examine metropolitan differences in the covariates. All analyses were weighted using weights provided by the Census Bureau to provide representative estimates at the state and MSA level for individuals aged 18 years and older.

## Results

Table 1 presents summary characteristics of survey respondents. Differences in ethnicity, unemployment and household income between the two Southern California MSA (LA-Anaheim and Riverside-San Bernardino) and the Northern California MSA (SF-Bay Area) are notable. Forty-three percent and 47 % of respondents in LA-Anaheim and Riverside-San Bernardino, respectively, were Hispanic compared with only 20 % in the SF-Bay Area which had higher percentages of Whites (43 %) and Asians (23 %). Compared with the SF-Bay Area, household income among respondents was lower in Riverside-San Bernardino and LA-Anaheim where 15 % had annual incomes of < $25 000, and only 18 and 25 %, respectively, had incomes higher than $100 000. In comparison, only 9 % of SF-Bay respondents had household incomes < $25 000 a year, while 44 % had incomes of more than $100 000 a year.

**Table 1:** Characteristics of study population by California geography; Census Household Pulse Survey, phase 1 (23 April–21 July 2020)*

	California	Los Angeles-Anaheim MSA	Riverside-San Bernardino MSA	San Francisco-Bay Area MSA
Unweighted *n*	76 008	22 240	15 540	20 095
Weighted Sample† (percentage of California sample)	100	35	13	12
Individual characteristics				
Female (%)	51	49	50	50
Median age (years)	46	44	46	46
Race/ethnicity (%)				
White	39	31	34	43
Black	6	6	8	8
Hispanic	36	43	47	20
Asian	14	17	7	23
Bachelor’s degree or higher (%)	32	32	19	47
Pre-pandemic food insufficient (%)	9	10	12	8
Recently lost employment income (%)	55	60	59	49
Unemployed (%)	34	38	38	27
Household characteristics				
Household income (%)				
< $25K	15	17	16	8
$25K–$50K	26	28	31	16
$50K–$100K	27	26	31	22
$100K+	33	30	22	54
Median household size	3	4	4	3
Median number of kids	2	2	2	2
Survey week (%)				
Week 1 (23 April–5 May)	8·3	8·7	9·0	7·8
Week 2 (7 May–12 May)	8·3	8·7	8·7	9·5
Week 3 (14 May–19 May)	8·3	8·8	8·1	9·7
Week 4 (21 May–26 May)	8·3	8·3	8·4	8·8
Week 5 (28 May–2 June)	8·3	8·7	8·2	8·4
Week 6 (4 June–9 June)	8·3	8·1	8·7	7·4
Week 7 (11 June–16 June)	8·3	8·6	7·7	8·1
Week 8 (18 June–23 June)	8·3	8·1	8·2	8·0
Week 9 (25 June–30 June)	8·3	8·1	7·9	8·6
Week 10 (2 July–7 July)	8·3	8·2	8·3	7·8
Week 11 (9 July–14 July)	8·3	7·5	8·7	8·0
Week 12 (16 July–21 July)	8·3	8·2	8·3	8·0

MSA, metropolitan statistical area.

* Weighted data provide representative estimates at the MSA and state levels.

† Proportions for MSA do not add to 100 % because additional households were surveyed in the state of California outside of the three MSA highlighted in these analyses.

Education levels were lowest among respondents in Riverside-San Bernardino where only 19 % had a college degree compared with 32 % in LA-Anaheim and 47 % in SF-Bay Area. Similar to the data reported above, during the 12 weeks of the survey, respondents in LA-Anaheim and Riverside-San Bernardino experienced higher unemployment rates than respondents in the SF-Bay Area (38 %, 38 % *v*. 27 %, respectively). Pre-pandemic food insufficiency rates were highest among respondents in Riverside-San Bernardino (12 %) compared with 10 % in LA-Anaheim and 8 % in SF-Bay Area.

**Fig. 1:**
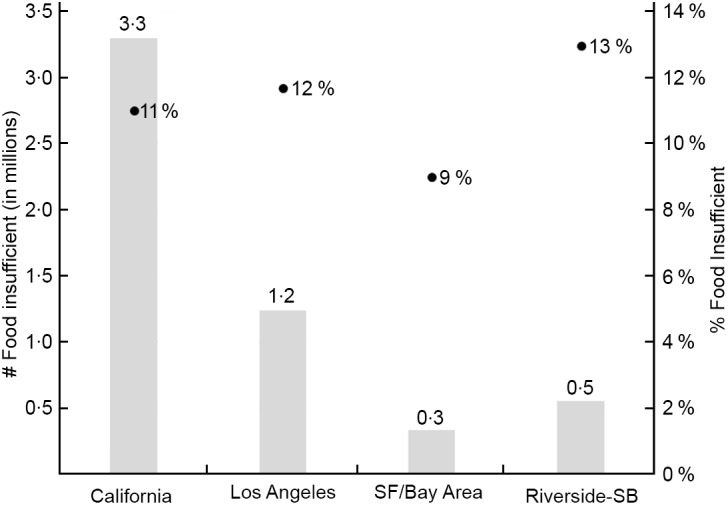
Number and percentage of adults in food insufficient households in California metropolitan statistical areas (MSA), Census Household Pulse Survey, phase 1 (23 April–21 July 2020)* *Data are weighted to provide representative estimates for adults aged 18 years and older living in households at the MSA and state level. MSA include San Francisco–Oakland–Berkeley, CA (SF/Bay Area), Los Angeles–Long Beach–Anaheim, CA (Los Angeles) and Riverside–San Bernardino–Ontario, CA (Riverside-SB). 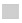, # food insufficient; 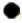, % food insufficient.

Over the initial months of the COVID-19 crisis from April to July 2020, more than three million California adults reported experiencing household food insufficiency, an increase of 22 % from the pre-pandemic rate of 9 % (Fig. 1). Consistent with the pattern observed prior to the COVID-19 crisis, food insufficiency rates were highest in Riverside-San Bernardino (13 %) closely followed by LA-Anaheim (12 %), and lowest in the SF-Bay Area (9 %). Given that LA-Anaheim is the second most populous MSA in the nation, the actual number of adults experiencing household food insufficiency in LA-Anaheim was 4 times and 2·5 times that in SF-Bay Area and Riverside-San Bernardino, respectively. Table 2 presents food insufficiency rates by individual and household characteristics for California and each MSA. For California, rates of food insufficiency were highest among respondents who were Black, Hispanic, previously food insufficient, unemployed and lower income. When comparing across the three MSA, SF-Bay Area respondents generally reported lower or similar rates of food insufficiency compared with those in the other two MSA. There were two exceptions to this finding: lower income and Black respondents in the SF-Bay Area were much more likely to report household food insufficiency than those in LA-Anaheim and Riverside-San Bernardino.

**Table 2:** Food insufficiency rates by California geography, Census Household Pulse Survey, phase 1 (23 April–21 July 2020)*

	California	Los Angeles-Anaheim MSA	Riverside-San Bernardino MSA	San Francisco-Bay Area MSA
Unweighted *n*	76 008	22 240	15 540	20 095
Overall food insufficiency (%)	11	12	13	9
Individual characteristics (%)				
Female	11	11	13	9
Age 65 or older	5	5	7	4
Race/ethnicity				
White	7	6	10	3
Black	22	20	22	32
Hispanic	17	18	16	18
Asian	6	6	8	5
Bachelor’s degree or higher	3	4	6	2
Pre-pandemic food insufficient	77	74	77	78
Recently lost employment income	16	17	19	14
Unemployed	21	21	21	21
Household characteristics (%)				
Household income				
< $25K	28	27	26	32
$25K–$50K	16	16	17	21
$50K–$100K	8	9	8	7
$100K+	1	2	2	1
School-age children at home	14	15	14	16

MSA, metropolitan statistical area.

* Weighted data provide representative estimates at the MSA and state levels.

### California

Table 3 presents the results of the multivariate regression analysis for California and for each of the MSA. Among all California respondents, pre-pandemic food insufficiency was by far the strongest correlate of food insufficiency during the COVID-19 crisis. Adults experiencing household food insufficiency prior to COVID-19 were forty times more likely to be food insufficient during the 12 weeks of the survey. Among adults that experienced household food insufficiency during COVID-19, almost 80 % were food insufficient prior to the pandemic.

**Table 3:** Multivariate logistic regression models estimating food insufficiency by California geography, Census Household Pulse Survey, phase 1 (23 April–21 July 2020)†

	California		Los Angeles-Anaheim MSA		Riverside-San Bernardino MSA		San Francisco-Bay Area MSA	
	OR	95 % CI	OR	95 % CI	OR	95 % CI	OR	95 % CI
Unweighted *n*	76 008		22 240		15 540		20 095	
Individual characteristics								
Female	0·95	0·78, 1·16	0·95	0·69, 1·30	0·95	0·70, 1·31	1·03	0·68, 1·55
Age	1·00	1·00, 1·00	1·00	1·00, 1·00	1·00	1·00, 1·00	1·00	1·00, 1·00
Age^2^	1·03	0·99, 1·07	1·04	0·98, 1·11	1·03	0·98, 1·08	0·98	0·91, 1·05
Race/ethnicity (Ref: White)								
Black	1·33	0·94, 1·88	1·65	0·88, 3·12	1·47	0·87, 2·48	2·83	1·51, 5·33
Hispanic	0·94	0·73, 1·21	1·13	0·75, 1·71	0·78	0·55, 1·12	1·23	0·74, 2·07
Asian	0·68	0·52, 0·88	0·67	0·42, 1·08	0·60	0·32, 1·15	1·17	0·74, 1·87
Bachelor’s degree or higher	0·50*	0·42, 0·60	0·54*	0·40, 0·74	0·57*	0·37, 0·89	0·43*	(0·29, 0·65
Pre-pandemic food insufficient	40·18*	32·19, 50·16	33·75*	23·40, 48·68	46·91*	33·58, 65·52	55·33*	34·30, 89·25
Recently lost employment income	2·16*	1·68, 2·77	2·40*	1·65, 3·50	2·56*	1·73, 3·80	2·36*	1·59, 3·50
Unemployed	1·87*	1·53, 2·30	2·07*	1·51, 2·85	1·52*	1·14, 2·04	1·30*	0·88, 1·93
Household characteristics								
Household income (Ref: > $100K)								
< $25K	2·52*	1·86, 3·42	2·06*	1·22, 3·46	2·19*	1·41, 3·39	3·63*	1·96, 6·74
$25K–$50K	1·73*	1·30, 2·29	1·72*	1·04, 2·86	1·52*	1·01, 2·28	3·39*	2·07, 5·54
$50K–$100K	1·28	0·98, 1·67	1·53	0·95, 2·46	1·10	0·71, 1·71	1·52	0·84, 2·72
Household size	1·11*	1·04, 1·18	1·05	0·95, 1·16	1·14	1·00, 1·29	1·00	0·90, 1·11
Number of kids	0·94	0·85, 1·04	1·05	0·90, 1·22	0·82	0·69, 0·97	1·05	0·87, 1·26
Metropolitan area (Ref: Rest of California)								
Los Angeles-Anaheim MSA	0·98	0·77, 1·25						
Riverside-San Bernardino MSA	1·02	0·80, 1·29						
San Francisco-Bay Area MSA	0·92	0·71, 1·19						
Survey Week (Ref: week 1)								
Week 2 (7 May–12 May)	1·21	0·68, 2·17	1·32	0·45, 3·88	1·35	0·64, 2·85	0·98	0·35, 2·74
Week 3 (14 May–19 May)	1·15	0·72, 1·85	1·49	0·70, 3·17	1·56	0·85, 2·83	0·73	·28, 1·92
Week 4 (21 May–26 May)	1·12	0·69, 1·84	1·26	0·64, 2·49	0·82	0·37, 1·79	0·26	0·11, 0·65
Week 5 (28 May–2 June)	1·24	0·78, 1·97	0·85	0·43, 1·66	1·37	0·78, 2·40	1·21	0·54, 2·70
Week 6 (4 June–9 June)	1·11	0·65, 1·92	1·18	0·53, 2·61	1·08	0·60, 1·97	0·86	0·38, 1·97
Week 7 (11 June–16 June)	1·29	0·80, 2·07	1·11	0·55, 2·22	1·40	0·73, 2·70	1·16	0·50, 2·67
Week 8 (18 June–23 June)	1·23	0·75, 2·00	0·90	0·46, 1·79	1·16	0·50, 2·69	0·72	0·24, 2·15
Week 9 (25 June–30 June)	1·33	0·85, 2·07	1·40	0·69, 2·82	1·03	0·60, 1·76	1·19	0·56, 2·49
Week 10 (2 July–7 July)	1·16	0·70, 1·92	1·18	0·55, 2·52	0·75	0·36, 1·57	0·86	0·40, 1·84
Week 11 (9 July–14 July)	1·24	0·79, 1·97	1·35	0·66, 2·76	1·42	0·76, 2·67	0·58	0·25, 1·36
Week 12 (16 July–21 July)	1·64*	1·04, 2·59	1·40	0·67, 2·90	2·51*	1·38, 4·56	1·05	0·46, 2·41

MSA, metropolitan statistical area; Ref, reference group.

* *P* < 0·05.

† Weighted analyses provide representative estimates at the MSA and state levels.

Controlling for pre-pandemic food insufficiency, income remained a statistically significant predictor of food insufficiency during COVID-19 and showed a dose–response relationship. The lowest income households (< $25k) were 2·5 times more likely to be food insufficient than households with incomes > $100k. Food insufficiency also remained high among households in the next income group; households with incomes between $25 000 and $50 000 were 1·7 times more likely to be food insufficient compared to households with incomes > $100 000. These two income categories included households in the bottom two income quintiles in California^(17)^.

Respondents with a Bachelor’s degree or higher were half as likely to experience household food insufficiency during the COVID-19 crisis as those with lesser education, an association independent of income and race/ethnicity. Employment status was also highly associated with food insufficiency. Adults who recently lost employment income were more than twice as likely to be living in food insufficient households as those who did not experience recent loss of income. Sixteen percent of adults in California who lost employment income, and 21 % of unemployed adults in California reported being food insufficient. For the state of California, the odds of experiencing household food insufficiency among those who were unemployed were 1·9 times higher than those who were employed. Being Black or Hispanic was not associated with increased odds, while being Asian was associated with decreased odds (OR: 0·68) of household food insufficiency compared with Whites, controlling for other socio-demographic characteristics. Survey week was included as a covariate to assess whether food insufficiency increased over the 12 weeks relative to week 1 (23 April–5 May 2020). As shown in Table 3, the odds of household food insufficiency increased each week but did not reach statistical significance until week 12 (16–21 July) when it was 1·6 times that in week 1. The MSA fixed effects were not statistically significant.

### Regional differences

Controlling for other factors, regional differences in the predictors of food insufficiency remained. While food insufficiency rates were much lower in the SF-Bay Area than in LA-Anaheim and Riverside-San Bernardino, disadvantaged households in the SF-Bay Area were more likely to be food insufficient. For example, SF-Bay Area households that were food insufficient prior to the COVID-19 crisis were fifty-five times more likely to be food insufficient during the crisis than food sufficient households, compared with forty times more likely statewide. Similarly, SF-Bay Area households in the bottom two income categories were about twice as likely to be food insufficient than their counterparts in LA-Anaheim and Riverside-San Bernardino. In fact, in the SF-Bay Area, adults in the second income category ($25–50k) were only slightly less likely to be food insufficient than those in the lowest income category, likely reflecting the high cost of living in this metropolitan area. Finally, the SF-Bay Area was the only metropolitan area in which being Black – controlling for other characteristics – was associated with higher rates of food insufficiency. In particular, Black households living in the SF-Bay Area were 2·8 times more likely to be food insufficient than non-Hispanic White households. In comparison, Black households in Riverside-San Bernardino and LA-Anaheim were not more likely to be food insufficient than non-Hispanic White households after controlling for other characteristics.

## Discussion

Many of the findings are consistent with those of other studies of food insufficiency as well as food insufficiency in the context of the COVID-19 crisis^(7,8)^. For California and all three metropolitan areas, adults with the greatest risk of household food insufficiency during the initial months of the COVID-19 crisis were those who were previously food insufficient. Respondent’s employment status, low household income (< $25 000 and $25 000–$50 000) and not having a Bachelor’s degree were also risk factors for food insufficiency for the state and for each of the three MSA.

We noted that food insufficiency varied minimally from week to week except towards the end of phase 1 of the survey. In week 12 (16–21 July 2020), which coincided with the expiration of unemployment benefit enhancements as part of the federal CARES Act, the odds of experiencing household food insufficiency were 1·67 times higher than in week 1 of the survey for California. This difference was amplified for Riverside-San Bernardino. For the other two MSA, household food insufficiency rates changed minimally over the 12 weeks.

Our most notable finding was the regional differences in the effects of socio-demographic factors on household food insufficiency. In particular, the associations of socio-demographic factors with food insufficiency were magnified for SF-Bay Area households. For example, Black households residing in SF-Bay Area were more likely to suffer from food insufficiency during the COVID-19 crisis than Black households residing in the Southern California MSA, adjusting for income and education. Low-income households in the SF-Bay Area also had higher odds of food insufficiency (3·63) than low-income households in LA-Anaheim and Riverside-San Bernardino (2·06 and 2·19, respectively). Geographic differences in food insufficiency experienced by Black and low-income households may be partially explained by differences in the benefits received from the CARES Act, and/or local responses to the pandemic. Rates of stimulus receipt from the CARES Act in our CHHPS study population differed by metropolitan area, with 83 and 87 % of LA-Anaheim and Riverside-San Bernardino residents receiving the stimulus, respectively, and just 72 % of SF-Bay Area residents receiving the stimulus (data not shown).

As we noted previously, income inequality and the cost of living are high in the SF-Bay Area. Studies suggest an association between inequality and a host of negative effects, including adverse health outcomes. Income inequality has both direct and indirect effects^(18,19)^. It directly produces disparities in purchasing power for goods such as fresh produce, which are likely compounded in high-cost metropolitan areas with expensive housing^(20,21)^. Income inequality also may have indirect effects on food insufficiency through social factors that alter the relationship between individuals and the food environment. For example, it may influence social bonds among family members and neighbours, the distribution of political influence and the allocation of resources that affect the accessibility and reach of social service programmes including food assistance programmes^(22)^.

Among households with children, geographic differences in participation in federal child nutrition programmes, such as the Special Supplemental Nutrition Program for Women, Infants and Children and the School Meals Program or in the implementation of these programmes during the COVID-19 crisis may partially explain metropolitan disparities in food insufficiency. Other scholars have observed that simultaneous participation in federal nutrition programmes, such as Women, Infants and Children and SNAP, is associated with lower risk of household food insecurity^(23)^. Differences in the implementation of these two programmes, for example, in outreach activities or in the application process, may explain varying coverage rates (defined as the percentage of those eligible who are enrolled in the programme).

Local responses to the waivers and flexibilities provided to school meals and child nutrition programmes under the Families First Coronavirus Response Act, passed in March 2020, varied considerably. These waivers and flexibilities allowed for the combination of food service operations from multiple entities to serve meals at a centralised location during school closures. Some local school districts provided free meals to all students while some others such as the San Francisco Unified School District provided free meals to students and their siblings^(24)^. Notably, the Los Angeles Unified School District, one of the largest in the nation, responded by providing free food at ‘Grab and Go’ food centres not only to students but to community members as well^(25)^. In addition, the presence of an active local food policy council may have supported efforts of the Los Angeles Regional Food Bank to widely distribute food through community-based organisations including faith-based entities^(26,27)^. It is likely that having an established infrastructure and network prior to the pandemic may have allowed some school districts to respond more effectively to the sudden increases in food insecurity brought about by the pandemic.

Interestingly, the number of children in the household was inversely associated with the odds of food insufficiency in Riverside-San Bernardino where the average household size is 3·3 compared with 3·0 in LA-Anaheim and 2·7 in SF-Bay Area. Because SNAP benefits increase with household size, it is possible that the lower odds of food insufficiency in Riverside-San Bernardino among low-income households may be partially due to receipt of higher SNAP benefits. Unfortunately, this phase of the CHHPS did not gather information on SNAP participation. The reach of SNAP, the largest federal food assistance programme which serves about 35·7 million Americans and costs about $60 billion annually^(28)^, varies widely among states. SNAP in California (also known as ‘CalFresh’) has one of the lowest coverage rates (defined as the percentage of the population who is eligible to participate in the programme) in the nation averaging about 71 % in 2017 compared with 84 % for the USA.^(29)^ Further, coverage rates vary widely among the fifty-eight counties in California where, unlike most other states, counties are given considerable autonomy in administering the SNAP programme; this may account for differences in reach and application rates. In 2018, SNAP coverage rates for SF-Bay Area counties ranged from 36 to 71 %, which were considerably lower than those for the other two MSA^(30)^. Coverage rates for counties making up the LA-Anaheim and Riverside-San Bernardino MSA ranged from 59 to 74 % and 68 to 92 % respectively, with San Bernardino County recording one of the highest coverage rates in the state^(30)^.

Data from phase 2 of the CHHPS (19 August–26 October 2020) show similar regional differences in SNAP participation. Starting in this second phase of data collection, the Census Bureau asked respondents about SNAP participation. From mid-August through September 2020, 32 and 35 % of respondents in households earning < $25 000 a year received SNAP in Riverside-San Bernardino and LA-Anaheim MSA, respectively, compared with only 27 % in the SF-Bay Area. This regional difference in coverage rates is even wider among households earning between $25K and $50K: 22 % received SNAP in San Bernardino-Riverside, 19 % in LA-Anaheim and just 12 % in the SF-Bay Area. Hence, higher food insufficiency in the SF-Bay Area among vulnerable population groups may partially reflect less effective SNAP administration and outreach in that region, factors that are likely to influence SNAP participation. Some of the lowest coverage rates for SNAP in the country are reported by counties in the SF-Bay Area. Understanding the reasons for these low coverage rates in this generally affluent region in the country may help address health disparities in regions where there are widening income disparities between the ‘haves’ and ‘have-nots’.

While the CHHPS data and the current analysis were useful in tracking food insufficiency during the course of the pandemic, they do have limitations. First, the CHHPS was administered online, so populations without access to the internet may be under-represented in the study sample. Second, the few questions on food insufficiency do not fully align with the larger concept of ‘food insecurity’ used by the US Department of Agriculture, likely underreporting the percentage of households that have enough food to lead healthy and active lives. Third, the first phase of data collection did not include information on the receipt of food assistance programme benefits such as from SNAP. Finally, the sampling design did not allow for analyses by counties, which in the case of California would have allowed for more targeted monitoring of food insecurity and the impact of established food assistance programmes such as SNAP.

## Conclusions

Our findings show regional differences in the association of various socio-demographic factors with food insufficiency risk. In particular, disadvantaged households in the SF-Bay Area – where income and educational levels are higher, but income inequality and cost of living are also higher – seem to be at higher risk for food insufficiency. Existing federal food assistance programmes can play an important role in addressing food insecurity in times of crisis, especially for low-income households. Having the administrative structure to effectively implement financial assistance programmes, SNAP and other food assistance programmes and quickly respond to changes in policy provisions is essential for mitigating both the immediate effects of a crisis such as the COVID-19 pandemic and the longer-term effects of structural inequities on food security. Food insecurity increases the risk of obesity and diet-related non-communicable diseases over the life-course^(31)^, and finding ways to increase the reach of SNAP and other federal assistance programmes during this pandemic may help to decelerate widening disparities in health and perhaps even reverse its course.

## Supplementary material

For supplementary material accompanying this paper visit http://dx.doi.org/10.1017/S1368980021001889. click here to view supplementary material
